# Soil nutrients, enzyme activities, and bacterial communities in varied plant communities in karst rocky desertification regions in Wushan County, Southwest China

**DOI:** 10.3389/fmicb.2023.1180562

**Published:** 2023-06-14

**Authors:** Lan Gao, Weihan Wang, Xingyu Liao, Xing Tan, Jiaxing Yue, Wen Zhang, Jiaojiao Wu, J. H. Martin Willison, Qiuling Tian, Yun Liu

**Affiliations:** ^1^College of Resources and Environment, Southwest University, Chongqing, China; ^2^College of Computer and Information Science, Southwest University, Chongqing, China; ^3^School for Resource and Environmental Studies, Dalhousie University, Halifax, NS, Canada

**Keywords:** bacterial communities, plant communities, karst rocky desertification, co-occurrence network, soil properties

## Abstract

Vegetation restoration has become a common practice in karst rocky desertification (KRD) areas of southwestern China. The bacteria, which have made a connection between soil and plants, have been an important role in regulating the succession and restoration of karst vegetation. However, it is still unclear how soil bacterial communities and soil properties respond to natural vegetation restoration processes in karst areas. To address this gap, we investigated the soil nutrients, enzyme activity, and soil bacterial community among various plant communities, including farmland (FL), land with herbs only (SSI), herb-and-shrub land (SSII), woody thickets (SSIII), coniferous forest (SSIV), coniferous and broad-leaved mixed forest (SSV), and evergreen broad-leaved forest (SSVI). Our results found that SSII had the highest levels of soil organic matter, total nitrogen, available phosphorus, available nitrogen, sucrase, and β-glucosidase among all the plant communities. These results indicated that herb-and-shrub land have contributed to the rapid restoration of vegetation in KRD regions. FL exhibited the lowest levels of soil nutrients and enzyme activities, while showing the highest bacterial richness and diversity among all the plant communities. This suggested that appropriate human intervention can increase bacterial diversity and richness in the area. The predominant bacterial phylum also varied among the different plant communities, with *Actinobacteria* being the most abundant in SSI, SSII, SSIII, and SSIV, while *Proteobacteria* were the most abundant in SSV and SSVI. Furthermore, PCoA analysis demonstrated significant changes in the soil bacterial community structure, with SSI, SSII, SSIII, and SSIV had shared similar structures, while SSV and SSVI had comparable structures. As for soil characteristics, total phosphorus (TP) and total potassium (TK) were the primary factors affecting the soil bacterial community. SSV and SSVI had the most complex bacterial networks and were more stable than other groups. The genera *Ktedonobacter*, *norank_f_Anaerolineaceae*, and *Vicinamibacter* had the highest betweenness centrality scores and were identified as keystone genera in the co-occurrence network in KRD areas. In summary, our results have demonstrated that herb-and-shrub can promote community succession and increase soil nutrient levels in KRD regions.

## 1. Introduction

Karst ecosystems are considered as one of the most vulnerable terrestrial ecosystems characterized by a slow soil formation process (Zhao et al., 2017; Tang et al., 2018). As a result, unwise and over-intensive land use, such as deforestation, grazing, and farming, can easily lead to karst rocky desertification (KRD) in fragile karst geo-ecological environments (Bai et al., 2013). KRD is a global issue that occurs in most karst areas, including the European Mediterranean, Southeast Asia, and Southwest China, posing a threat to the long-term sustainability of these environments (Duringer et al., 2012; Salvati, 2014; Jiang et al., 2014). Ecological degradation has been one of the most important consequences caused by KRD, which is often characterized by desert-like landscapes, loss of endemic biomass, the decline in soil quality and biodiversity, droughts, and floods with greater frequency and severity (Ford and Williams, 2011; Yan and Cai, 2015). Soil degradation and vegetation destruction have exacerbated rocky desertification, making it the main ecological disaster that hinders the economic development of Southwest China (Chen et al., 2011; Zhang et al., 2021).

Vegetation restoration is widely recognized as an effective approach to mitigate soil erosion and promote vegetation growth, and has been extensively employed against KRD (Yan et al., 2020). Forest restoration can be roughly divided into two categories, including the restoration of planted forests with limited plant species and that of natural forests. Since natural forests can better protect biodiversity with ecosystem functions such as surface carbon storage, and soil and water conservation, related natural forest restoration is a better way to restore vegetation in KRD areas (Hua et al., 2022). However, mountain areas in Southwest China that suffered from severe desertification would take a long time to restore their natural forests (Jiang et al., 2014). Thus, artificial efforts like afforestation are necessary to accelerate the restoration process, so that the water and soil could be maintained to control the rocky desertification (Jiang et al., 2014; Tong et al., 2018). Previous research in related regions has revealed the phenomenon that though plant species resistant to drought and barren conditions were chosen, the restoration result could hardly meet the expectation (Cao et al., 2015). Therefore, it is far from enough to restore the vegetation in KRD areas using plant species with some specific features (Shen et al., 2018). More deep and comprehensive understandings of the natural forest restoration process are essential for positive artificial intervention (Tong et al., 2016). Specifically, details in the restoration process including composition changes in the ecosystems, mutual influence between vegetation and the environment should be carefully investigated so that we could know the factors which have promoted the positive succession of the communities. Since vegetation restoration changes the soil environment and affects the structure and diversity of soil bacterial community (Lu et al., 2022). Changes in vegetation types can greatly impact the microbial community structure through plant rhizosphere exudates and litter nutrient cycling (Zhu et al., 2012). Therefore, it is of vital importance to know about the change of soil properties and microbial communities during the restoration of natural vegetation. This knowledge can help to carry out effective and sustainable efforts for vegetation restoration in the KRD areas.

Soil microbes have important effects on plant health, soil productivity, and ecosystem functioning (Bardgett and Van Der Putten, 2014; Tang et al., 2018), while they can increase the soil nutrients and regulate the soil enzymes as well (Hu et al., 2016; Pan et al., 2018). Soil enzymes have been involved in the regulation of soil fertility, nutrient cycling, and carbon sequestration, making them important indicators of soil health and quality (Wei et al., 2011; Peng et al., 2020). As the dominant microbial taxa, bacteria are the most important decomposers of soil organic matter and litter, and they play an important role in promoting nutrient cycling and ecosystem processes regulation (Uroz et al., 2011; Ma et al., 2016). Bare land is a typical example (e.g., rocky desertification land), where bacteria have provided most of the available carbon source during the entire development process of biological soil crusts (BSCs), which is an indication of a reversal of desertification (Wang et al., 2021). Soil bacterial communities are extremely sensitive to environmental changes, and small changes in environmental factors can cause changes in their diversity and quantity, so they are often used as indicators of ecosystem changes (Sui et al., 2019). However, there has been little research on natural vegetation restoration in karst areas, with more focus on artificial vegetation restoration. For instance, Song et al. (2022) studied the effects of vegetation restoration for different cultivated pastures on soil bacterial communities in KRD regions. Lu et al. (2022) studied the effects of artificial vegetation restoration on soil in bacterial communities in karst areas. Although Li et al. (2021b) studied the changes of soil microbial communities during the succession of vegetation from bare rock to arbor forest. Wang et al. (2022) explored the effects of secondary succession on the composition and diversity of soil fungi and bacteria in karst areas. The high heterogeneity of habitat in karst areas has made it challenging to understand the impact of vegetation restoration on soil properties and bacterial communities. Although some studies have examined the effects of vegetation restoration on soil bacterial communities in KRD regions, more research is needed to further understand the impact of natural vegetation restoration on these communities (Guo et al., 2020). Thus, it is necessary to carry out more exploration of soil bacterial communities in KRD regions, so that we can better understand the vegetation restoration progress and plant community patterns and provide a theoretical basis for scientific development in artificial vegetation restoration (Delgado-Baquerizo et al., 2018; Krishna et al., 2020; Liang et al., 2020).

Therefore, we characterized the change in soil nutrients, enzyme activity, and bacterial community in varied plant communities in the KRD regions. Soil bacterial community was surveyed by deep sequencing of 16S hypervariable 3–4 regions (V3–4) and Illumina HiSeq platform. We expected that the soil properties and soil bacterial community compositions and structures would vary among different plant communities, and specific bacterial taxa would be associated with different vegetation types. In this paper, we aim to investigate the changes in soil properties and bacterial communities in different stages of natural vegetation restoration in the KRD area of Southwest China. We seek to understand the interactions between soil and plant communities. This knowledge can not only help to guide artificial vegetation restoration efforts, but also provide valuable information for the preservation of the unique ecosystem in the KRD region.

## 2. Materials and methods

### 2.1. Site description and sample collection

Wushan County is a typical KRD territory in Northeast Chongqing, which is highly susceptible to desertification (Qi et al., 2018) and about 70% of its land has been moderate and severe KRD areas (Qi et al., 2017). Thus, the study focuses on KRD regions in Wushan County of Chongqing, which is characterized by a typical subtropical monsoon humid climate with mild winter and spring, periodic droughts in summer between periods of monsoon rains, four distinct seasons, and high humidity for most time of a year. The average annual temperature and precipitation in the study area were 18.4°C and 1,041 mm, respectively.

**Figure 1:**
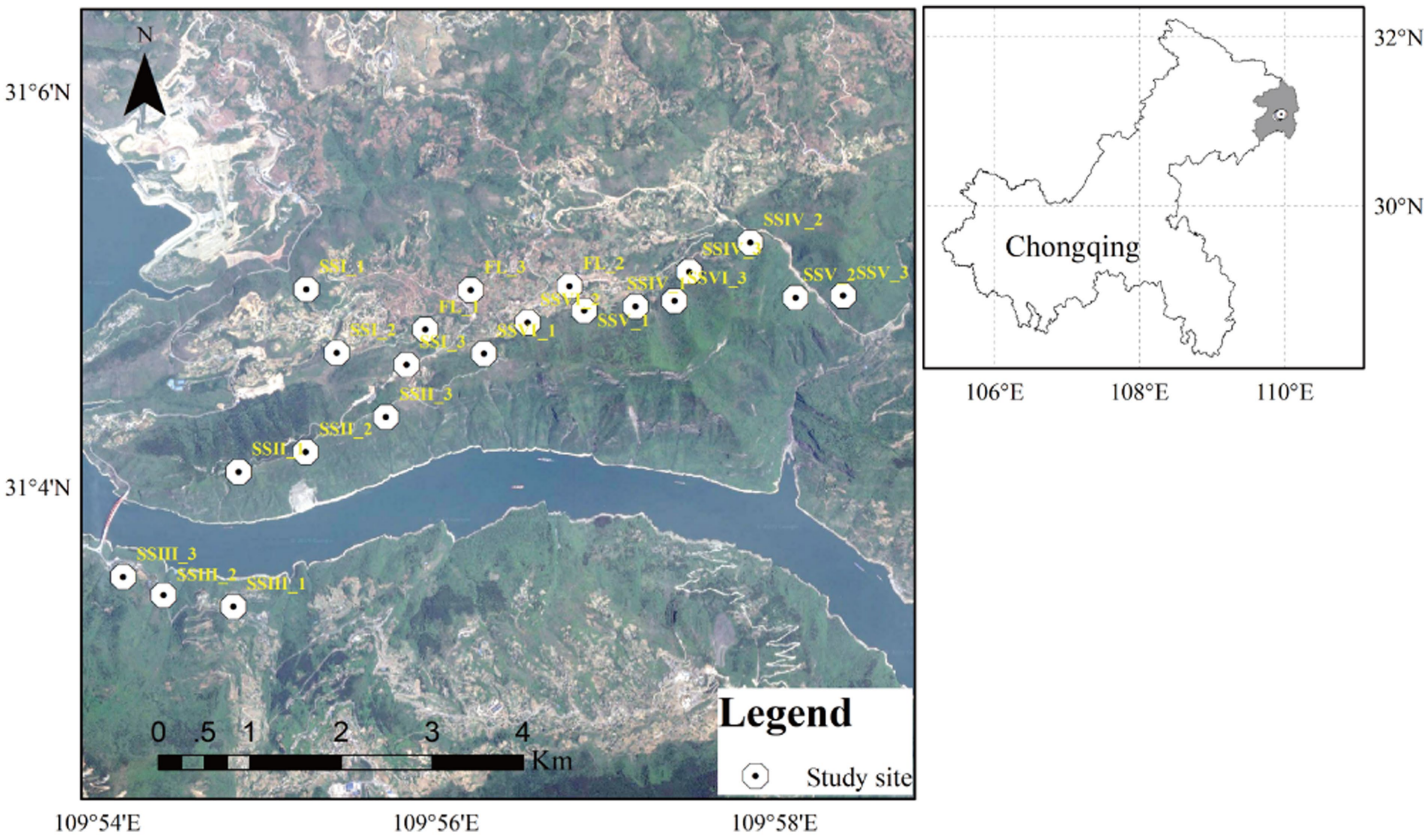
Map of the study area located in Northeast Chongqing. Twenty-one sampling sites (10 m × 10 m) were set, three for each study site in karst rocky desertification regions.

The major soil types were Xanthic ferrasols and Haplic luvisols, as classified by the Food and Agriculture Organization of the United Nations (FAO) guidelines (Gong, 2001). Six different plant community types were selected, including herbs only, herb-and-shrub land, woody thickets, conifer forests, conifer and broad-leaf mixed forests, and evergreen broad-leaf forests (SSI, SSII, SSIII, SSIV, SSV, and SSVI), along with nearby farmland (FL; Supplementary Table S1; Figure 1). Then the plant composition in each plant community was investigated (Supplementary Table S2), and the abundance of the typical plant species is shown in parentheses after each species according to the Clements abundance level (Supplementary Table S1).

Soil samples were collected in June 2018 using the S-shaped sampling method. Three 10 × 10 m quadrats (*n* = 21 in total) were randomly arranged in each plant community, with a distance of more than 50 m between each quadrat to exceed the spatial autocorrelation distance of microbial variables (Zhou et al., 2019). Within each quadrat, there were ten sampling points. After removing the humus and impurities on the soil surface, a 5 cm diameter soil drill was used to collect samples from the 0–10 cm mineral soil layer. Then all 10 subsamples were mixed into one composite sample to comprehensively represent the bacterial community. Collection tools were autoclaved before use. Approximately one-quarter of each sample was preserved in a sterilized centrifuge tube under liquid nitrogen environment. Then these samples were stored in a freezer at −80°C for 16 s rDNA analysis. The remaining fresh soil samples (~ 500 g) were air-dried indoors and passed through a 2 mm sieve for soil properties determinations.

### 2.2. Analyses of soil nutrients and enzyme activities

Soil pH (soil:water = 1:2.5, *W*/*V*) was measured by a pH meter (IQ Scientific Instruments, IQ150, CA), while soil organic carbon (SOC) was determined through the potassium dichromate oxidation method (Nelson and Sommers, 1982), and soil organic matter (SOM) was calculated by multiplying SOC values by 1.724 (Walkley and Black, 1934). Total phosphorus (TP) and total potassium (TK) were determined after extraction by melted-NaOH, colorimetry for P and flame photometry for K, respectively, (Olsen and Sommers, 1982)_._ Available phosphorus (AP) was determined by NaHCO_3_ solution (pH 8.5, 0.5 mol/L; Olsen and Sommers, 1982), and total nitrogen (TN) was determined by the semi-micro Kjeldahl method (Bremner and Mulvaney, 1982). Finally, available nitrogen (AN) was determined by the alkali-hydrolyzed nitrogen diffusion method (Lennox and Flanagan, 1982).

Five soil enzyme activities linked to C (β-glucosidase and sucrase), N (urease), P (alkaline phosphatase) cycling, and hydrogen peroxide (catalase) were analyzed. All soil enzymes are measured with the kits (Sino best biological technology Co., Ltd., Shanghai, China) following the instructions. For further details on soil enzyme activity analyses, refer to the description in the Supplementary materials.

### 2.3. DNA preparation and sequencing

DNA extraction was performed using the E.Z.N.A.^®^ soil DNA Kit (Omega Bio-tek, Norcross, GA, United States), and the concentration of the purified DNA was determined using a NanoDrop 2000 UV–vis spectrophotometer (Thermo Scientific, Wilmington, United States). The 16S rRNA V3–V4 hypervariable regions were amplified with primers 338F (5′-ACTCCTACGGGAGGCAGCAG-3′) and 806R (5′-GGACTACHVGGGTWTCTAAT-3′) by a thermocycler PCR system (GeneAmp 9,700, ABI, United States). PCR reactions were performed in triplicate 20 μL mixtures containing 4 μL of 5 × FastPfu Buffer, 2 μL of 2.5 mM dNTPs, 0.8 μL of each primer (5 μM), 0.4 μL of FastPfu Polymerase, 10 ng of template DNA, and 0.2 μL BSA. The mixture was then topped up to 20 μL with ddH_2_O. The resulting PCR products were extracted from a 2% agarose gel and purified with the AxyPrep DNA Gel Extraction Kit (Axygen Biosciences, Union City, CA, United States) and quantified using QuantiFluor™-ST (Promega, United States) according to the manufacturer’s protocol. The purified amplicons were pooled in equimolar amounts and paired-end sequenced (2 × 300) on an Illumina MiSeq platform (Illumina, San Diego, United States) according to the standard protocols by Majorbio Bio-Pharm Technology Co. Ltd. (Shanghai, China).

### 2.4. Statistical analysis

To classify the resulting sequences, operational taxonomic units (OTUs) were clustered with 97% similarity cutoff using UPARSE (version 7.1). Silva (SSU123) 16S rRNA database^1^ was used to classify the taxonomy of each gene sequence with a confidence threshold of 70%.

We calculated rarefaction curves and alpha-diversity with Mothur (version 1.30.2) and performed unweighted pair-group method with arithmetic means (UPGMA) clustering with QIIME (version 1.9.1). To explore the effects of soil factors on the dominant bacterial community, we used R software (version 3.5.1) for PCoA (Principal co-ordinates analysis), heatmap of Spearman’s correlation coefficients, and redundancy analysis (RDA).

The variance inflation factor (VIF) was calculated by SPSS (version 23.0) to determine the collinearity of different soil factors across large spatial distances, and the detailed protocols were reported in previous studies (Zhou et al., 2019). In subsequent analysis, we removed the environmental factors with VIF values higher than 10 (Zhou et al., 2019), including soil organic matter (SOM), total nitrogen (TN), and available phosphorus (AP). Linear discriminant analysis (LDA) effect size (LEfSe; Segata et al., 2011)^2^ was used to obtain and identify communities or species with significant differences among plant communities.

To reduce network complexity, we selected the top 200 OTUs of each site as representative operational taxonomic units (OTUs) and three groups of samples divided by clustering results for network analysis. Spearman’s correlation coefficient between two OTU was considered statistically robust if its value (*ρ*) was >0.6 with a corresponding value of *p* < 0.01 (Barberán et al., 2012; Ma et al., 2016). Visualization of networks and calculation of network topological properties were performed through the interactive platform Gephi (version 0.9.2).

To study the differences in soil nutrients and enzyme activities among different plant communities, we used one-way ANOVA and Duncan’s new multiple-range method for multiple comparisons in SPSS (version 23.0). The results were presented as the arithmetic means of three replicates with standard deviations (SDs).

### 2.5. Accession numbers

The raw reads were deposited into the NCBI Sequence Read Archive (SRA) database (Accession Number: SRP213812).

## 3. Results

### 3.1. Plant community composition, soil properties, and enzyme activities

We surveyed the plant communities in all study sites and identified 27 plant species (Supplementary Table S2). Evergreen broad-leaved forest (SSVI) had the most abundant species, followed by coniferous and broad-leaved mixed forest (SSV). Additionally, we observed that *Cotinus coggygria* var. *pubescens* was widely distributed in the KRD regions of Wushan County. A comparison of the distribution area between KRD and *C. coggygria* worldwide using data from the ipant database^3^ and Plants of the World Online (POWO, https://powo.science.kew.org/) revealed highly overlapped distribution areas (Supplementary Figure S1). Thus, *C. coggygria* may play a critical role in vegetation restoration in the KRD regions.

Soil properties varied among different plant communities with varying pH values. Soils in sites SSI, SSII, SSIII, and SSIV were weakly alkaline, while that of site FL, SSV, and SSVI were weakly acidic (Supplementary Table S3). Soil organic matter, total nitrogen, and available phosphorus decreased from site SSII to site SSV, while increased slightly in site SSVI. Available nitrogen decreased gradually from site SSII to site SSVI. Site FL had the lowest soil organic matter, total nitrogen, and available nitrogen content, while total potassium was significantly higher than that in other sites. Total phosphorus content was similar in sites SSI to SSIII and FL, but higher in site SSIV.

Alkaline phosphatase (AKP) and catalase (CAT) activity were considerably higher in sites SSI to SSVI than in FL, with few differences among other plant communities (Supplementary Table S4). The activity of sucrase and urease (UE) in FL was the lowest among all study sites. Urease was highest in site SSIII, and gradually decreased from site SSIII to site SSV with a slight increase in SSVI. Sucrase (SC) activity was highest in SSII, while β-glucosidase (β-GC) was the lowest in SSII and the highest in SSIV.

### 3.2. Alpha-diversity of the bacterial communities

1,360,625 Illumina sequences from bacteria were identified, with each sample containing between 45,790 and 73,960 sequences. Good’s coverage exceeded 98% for all sites, indicating sufficient sampling (Table 1). Rarefaction curves also confirmed the sufficiency of sequencing data (Supplementary Figure S2). Analysis of similarities (ANOSIM; Supplementary Figure S3) revealed differences among study sites (*r* = 0.9628, *p* = 0.001). Thus, it can be concluded that the study sites had significant statistical differences (*p* < 0.001). FL had the highest number of OTUs (Supplementary Table S5), and the bacterial richness (Chao and ACE index), while bacterial richness of SSI was the smallest and significantly lower than that of SSII, SSIII, SSIV, and SSVI (*p* < 0.05, Supplementary Table S5). Bacterial diversity (Shannon indices) of FL was much higher than that of SSI, SSII, SSIII, SSIV, and SSVI (*p* < 0.05), while the lowest diversity was observed in SSVI.

**Table 1:** Bacterial diversity and richness indexes of the varied plant communities.

Study sites	OTUs	Shannon	Simpson	ACE	Chao	Coverage (%)
FL	1969 ± 25	6.52 ± 0.05	0.004 ± 0.001	2220 ± 46	2248 ± 32	98.70%
SSI	1498 ± 64	5.94 ± 0.09	0.007 ± 0.001	1801 ± 60	1816 ± 82	98.74%
SSII	1768 ± 18	6.16 ± 0.01	0.006 ± 0.001	2085 ± 15	2093 ± 24	98.57%
SSIII	1703 ± 77	6.12 ± 0.10	0.005 ± 0.001	2070 ± 68	2086 ± 40	98.51%
SSIV	1706 ± 23	6.20 ± 0.04	0.005 ± 0.001	2026 ± 40	2057 ± 63	98.61%
SSV	1612 ± 136	6.04 ± 0.28	0.008 ± 0.003	1927 ± 114	1968 ± 120	98.68%
SSVI	1631 ± 89	5.74 ± 0.21	0.014 ± 0.004	1984 ± 80	2003 ± 43	98.54%

### 3.3. Bacterial community composition and structure

**Figure 2:**
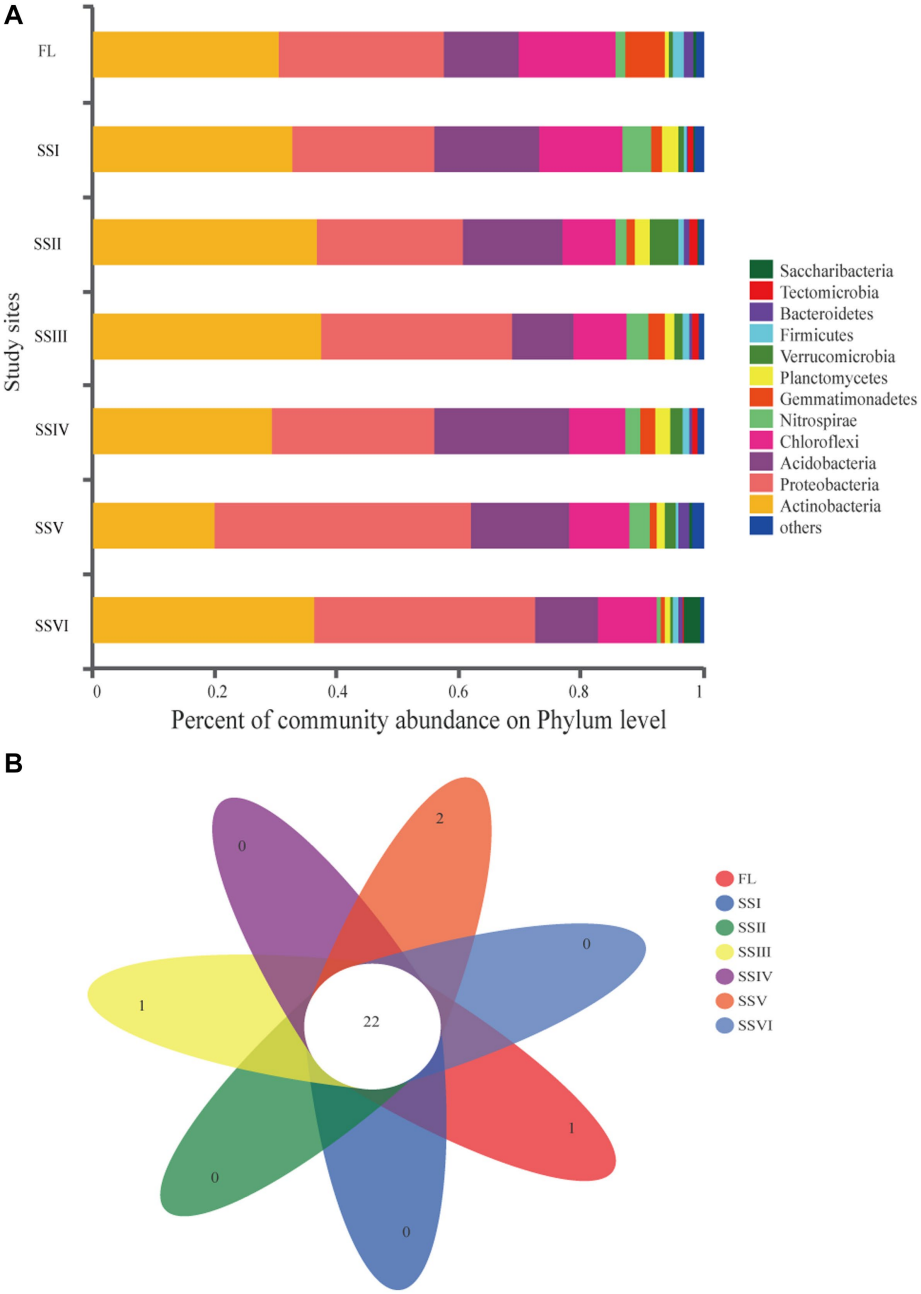
**(A)** Composition of soil bacterial community at Phylum level. **(B)** Venn diagram of exclusive and shared bacterial Phyla. Farm land (FL), land with only herbs (SSI), herb-and-shrub land (SSII), woody thickets (SSIII), coniferous forest (SSIV), coniferous and broad-leaved mixed forest (SSV), and evergreen broad-leaved forest (SSVI).

Figure 2A showed that *Actinobacteria*, *Proteobacteria*, *Acidobacteria*, and *Cleroflexi* were the dominant phyla across all study sites, accounting for over 80% of the clades. Other main phyla were *Nitrospirae* (0.52–4.74%), *Gemmatimonadetes* (0.75–6.47%), *Planctomycetes* (0.74–2.76%), *Verrucomicrobia* (0.48–4.70%), and *Firmicutes* (0.39–1.86%). The remaining 24 phyla with an abundance of less than 1% are classified as “others,” including *Tectomicrobia*, *Saccharibacteria*, *Latescibacteria*, *Cyanobacteria*, *Armatimonadetes*, etc. The Venn diagram showed 22 shared phyla among all study sites, while *FCPU426* of FL, *SBR1093* of SSIII, *Gracilibacteria*, and *Microgenomates* of SSV were unique to their respective sites (Figure 2B). *Actinobacteria* was dominant in SSI, SSII, SSIII, and SSIV, while *Proteobacteria* was the most abundant phylum in SSV and SSVI. Among the most abundant four phyla, the relative abundance of *Actinobacteria* significantly decreased in SSV compared to that in FL, SSI, SSII, SSIII, and SSVI (*p* < 0.05, Supplementary Figure S4). Bacterial composition at the class level was dominated by *Actinobacteria*, *AlphaProteobacteria*, and *Acidobacteria* across all plant communities, accounting for 20.03–37.46%,14.80–29.71%, and 10.23–21.98%, respectively. Other abundances are shown in Supplementary Figure S4.

**Figure 3:**
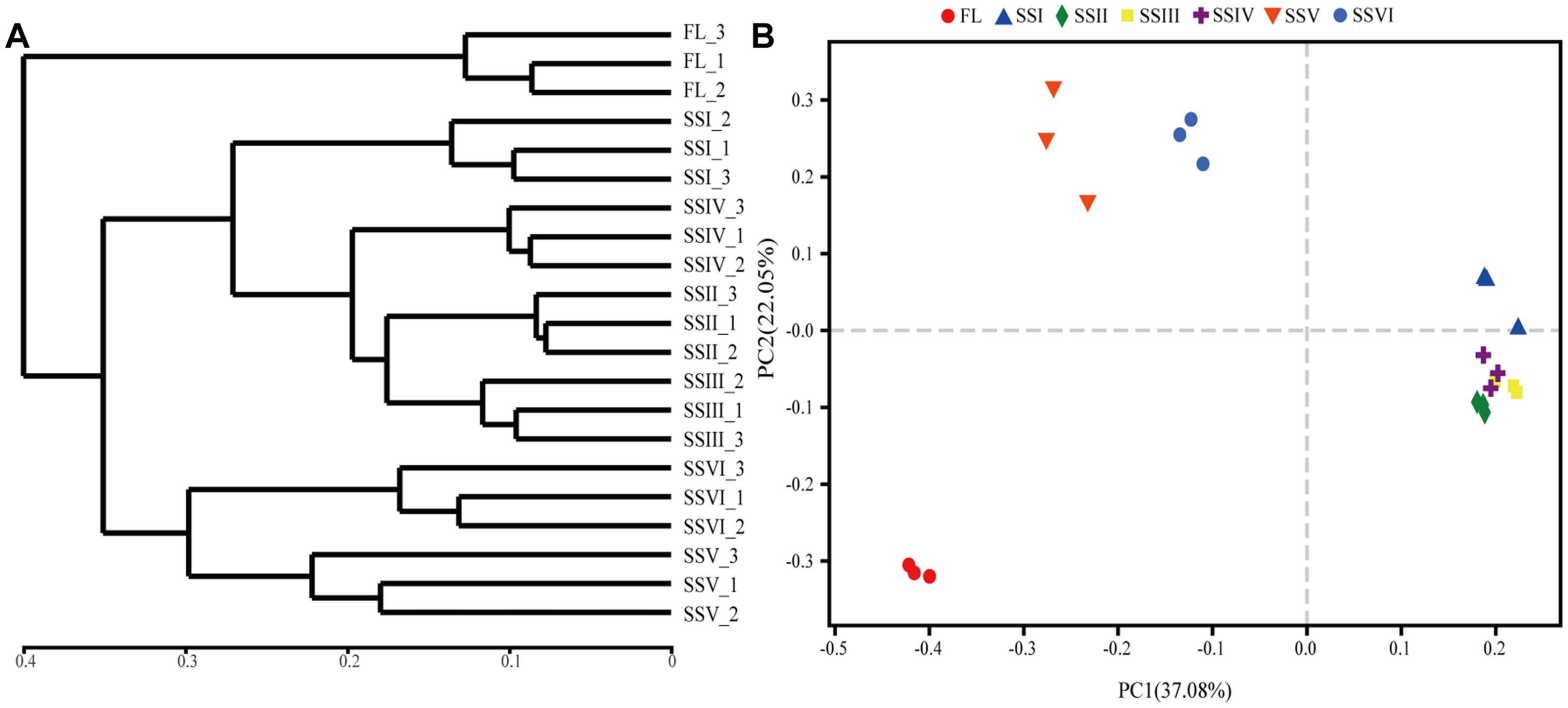
**(A)** Unweighted pair-group method with arithmetic means (UPGMA) clusters and **(B)** Principal co-ordinates analysis (PCoA) of bacteria community diversity in different study sites. Farm land (FL), land with only herbs (SSI), herb-and-shrub land (SSII), woody thickets (SSIII), coniferous forest (SSIV), coniferous and broad-leaved mixed forest (SSV), and evergreen broad-leaved forest (SSVI).

The UPGMA method and principal co-ordinates analysis (PCoA) were used to cluster the soil bacterial communities (Figure 3). The results identified three groups, with SSV and SSVI in one group and SSI, SSII, SSIII, and SSIV in another group. The FL sites were distinct from all other sites. PCoA results (*R*^2^ = 0.8786, *p* = 0.001, PERMANOVA) showed that the study sites were clearly distinguished, with the first and second principal components explaining 37.08 and 22.05% of the variation, respectively.

### 3.4. Indicator microbial taxa for each plant communities

**Figure 4:**
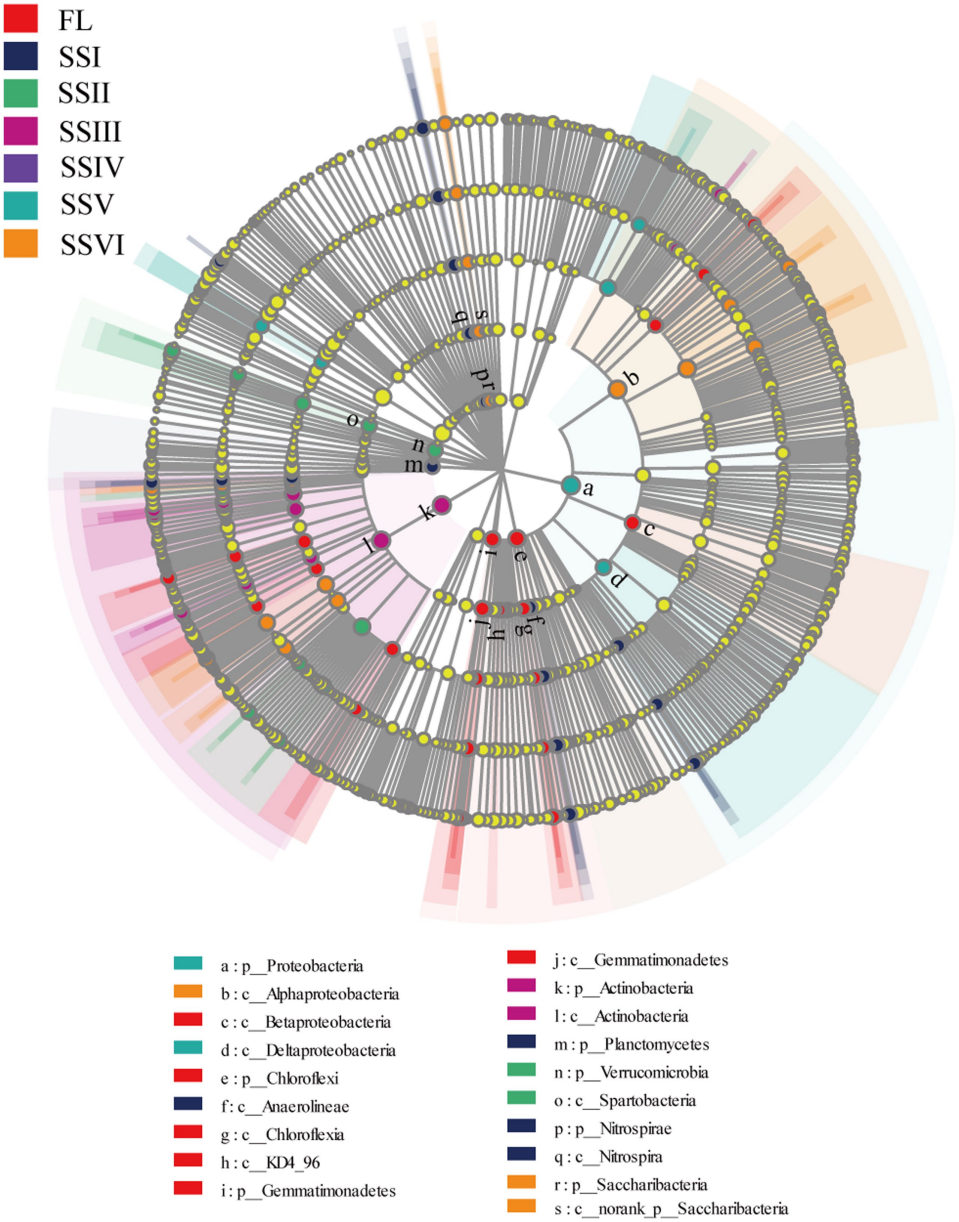
LEfSe analysis of different study sites.

Figure 4 shows 91 indicator bacteria among the study sites identified using LDA threshold of 4.0.The number of indicators for each site is as follows: FL (22), SSI (17), SSII (12), SSIII (13), SSV (7), and SSVI (20; Figure 4; Supplementary Figure S5). At the phylum level, *Chloroflexia* and *Gemmatimonadete*s were the indicator bacteria for FL, while *Nitrospirae* and *Planctomycetes* were indicators forSSI. *Verrucomicrobia*, *Actinobacteria*, *Proteobacteria*, and *Saccharibacteri* were the indicator bacteria for SSII, SSIII, SSV, and SSVI, respectively.

At the class level, four classes (*Beta Proteobacteria*, *Chloroflexi*a, *KD4_96*, and *Gemmatimonadete*s) were significantly enriched in FL, while *Nitrospira* and *Anaerolineae* were relatively enriched in SSI. *Spartobacteria, Actinobacteria*, and *Delta Proteobacteria* were enriched in SSII, SSIII, and SSV, respectively. Additionally, SSVI had more *Alpha Proteobacteria* and *norank_p_Saccharibacteria* compared to other classes. Furthermore, several bacterial taxa were overrepresented in plant communities, including *Chloroflexi* (phylum) for FL, *Solirubrobacterales* (order) for SSII, *Actinobacteria* (phylum), *Actinobacteria* (class), and *Gaiellales* (order) for SSIII, *Proteobacteria* (phylum) for SSV, and *Alphaproteobacteria* (class), *Rhizobiales* (order), *Xanthobacteraceae* (family), and *norank_f_Xanthobacteraceae* (genus) for SSVI (LDA score > 4.5).

### 3.5. Relationships among bacteria, soil, and enzyme activities

**Figure 5:**
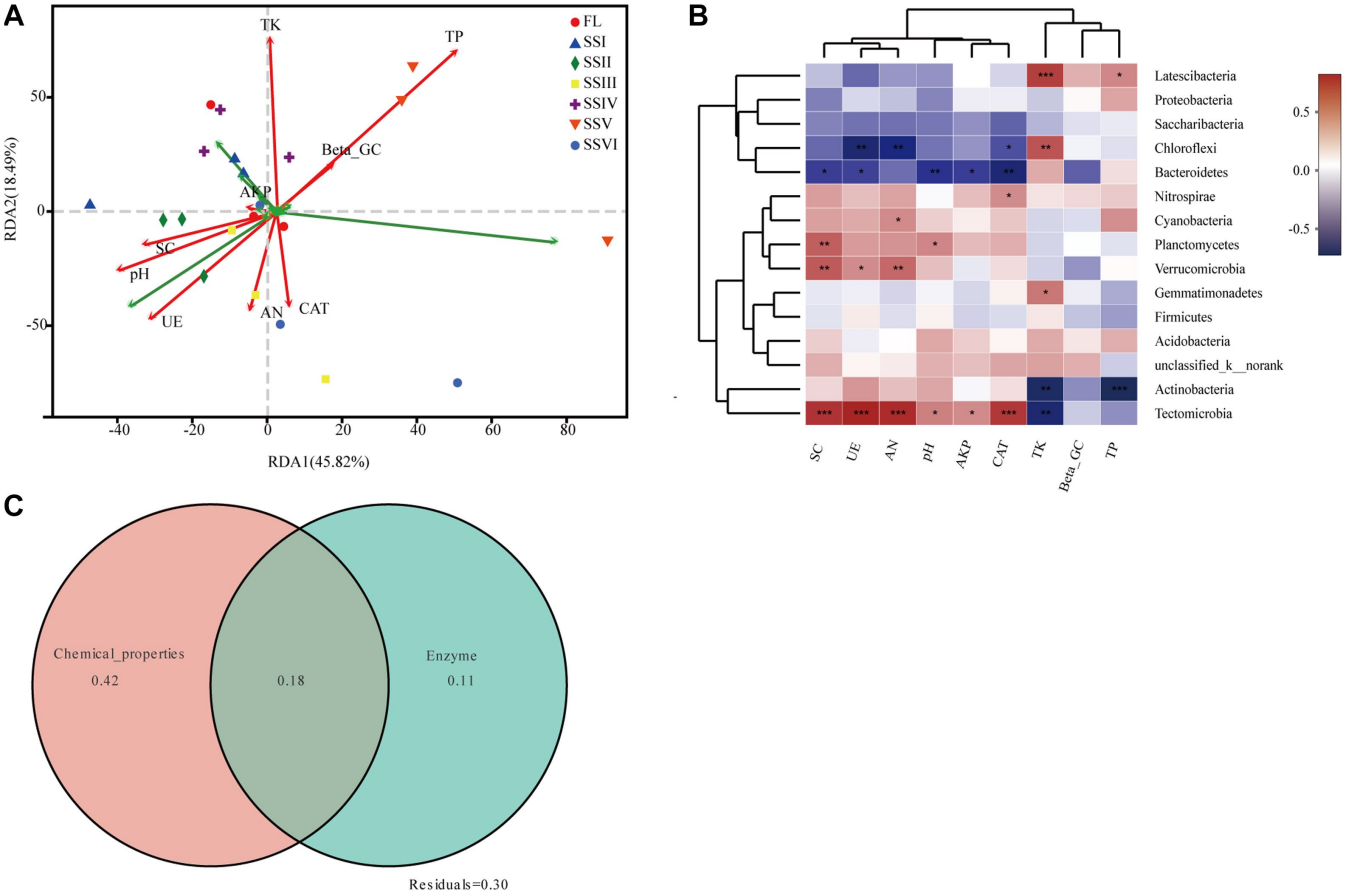
**(A)** Redundancy analysis (RDA) of bacterial communities (phylum level) and soil factors. **(B)** Heatmap of Spearman’s correlation coefficients between top 15 bacterial phyla and soil factors (^*^0.01 < *ρ* ≤ 0.05, ^**^0.001 < *ρ* ≤ 0.01, ^***^ρ ≤ 0.001). **(C)** Variation partitioning analysis of the bacterial community composition. The total variation was partitioned by soil chemical properties (pH, TP, TK, and AN) and Enzyme activity (β-GC, SC, UE, AKP, and CAT).

Redundancy analysis (RDA) was used to determine the relationships among soil bacteria, soil nutrients, and enzyme activities (Figure 5A). The first two axes explained 45.82 and 18.49% of the total variance, respectively. The results indicated that the primary soil factors driving soil bacterial community were total phosphorus (TP) and total potassium (TK; *p* < 0.05). Spearman correlation analysis demonstrated significant relationships between dominant bacterial phyla and soil factors (Figure 5B), including *Latescibacteria*, *Chloroflexi*, *Gemmatimonadete*, *Actinobacteria*, and *Tectomicrobia* with total potassium (TK), and *Latescibacteria* and *Actinobacteria* with total phosphorus (TP). Furthermore, variation partitioning analysis (Figure 5C) showed that soil chemical properties explained the largest variation in bacterial community composition, while soil enzyme activity had a comparatively small effect.

### 3.6. Co-occurrence network structure

**Figure 6:**
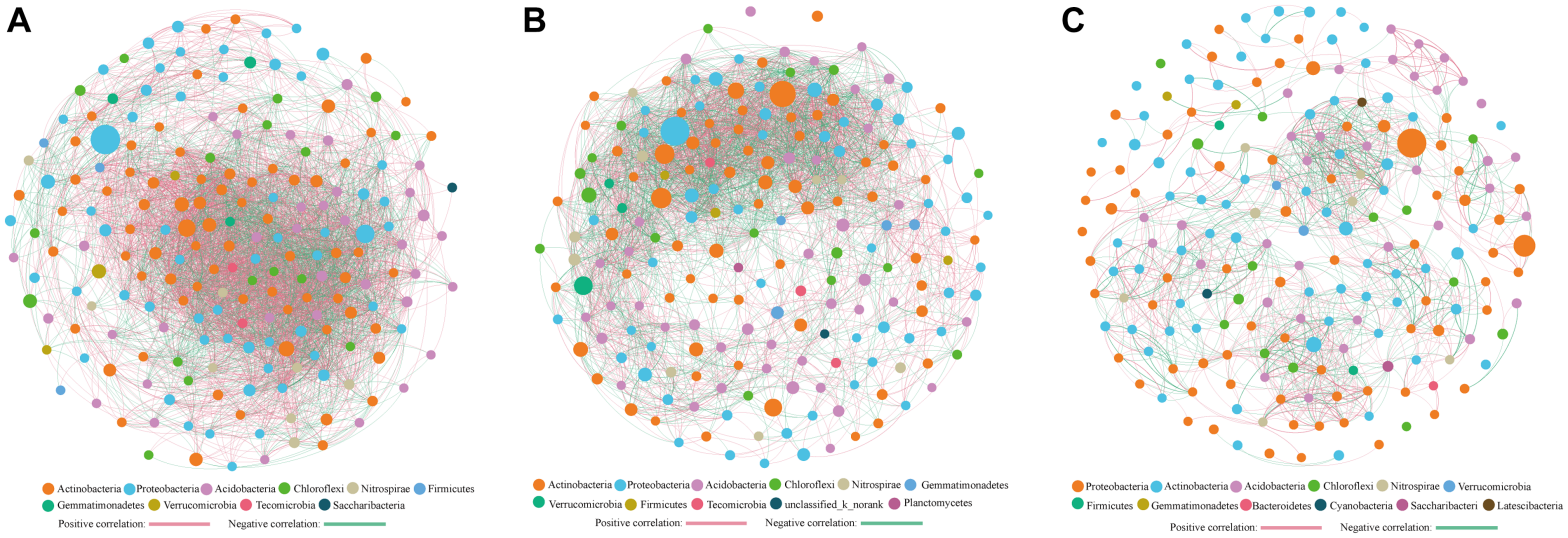
Co-occurring network of bacterial communities across **(A)** FL **(B)** SSI, SSII, SSIII, and SSIV **(C)** SSV and SSVI based on correlation analysis. The connections stand for a strong (spearman’ s *ρ* > 0.6) and significant (*p* < 0.01) correlations. The thickness of each edge is proportional to the *ρ*. Node colors and size represent the OUT taxa and the relative abundance of specific OTU at the phylum level.

Cluster analysis categorized the study sites into three groups (Figure 3), with co-occurrence network analysis revealing significant differences among them (Figure 6). The network’s topological properties were calculated to describe the complex relationships among the nodes. Group 3 had the most complex network, with an average clustering coefficient of 0.632, while groups 1 and 2 had average clustering coefficients of 0.591 and 0.607, respectively (Table 2). Group 3’s network was more stable than the others, with an average path length of 4.796 and a modularity index of 0.66. In contrast, group 1’s network was more susceptible to environmental interference, with an average path length of 2.059 and a modularity index of 0.26. Positive co-occurrence patterns predominated in the KRD regions, with the number of positive links accounting for 61.47, 52.21, and 53.3% of the total number of corresponding links in groups 1, 2, and 3, respectively. Meanwhile, the number of negative links accounted for 38.53, 47.79, and 46.70% of total links, respectively, indicating a more competitive correlation among bacteria in the network of group 2.

**Table 2:** Main properties of the co-occurrence network of FL (group 1), SSI, SSII, SSIII, and SSIV (group 2), SSV and SSVI (group 3).

Network indexes	Group 1 (FL)	Group 2 (SSI, SSII, SSIII, and SSIV)	Group 3 (SSV and SSVI)
Positive links	5,494	3,447	1,323
Negative links	3,443	3,156	1,160
Average clustering coefficient	0.591	0.607	0.632
Average path distance	2.059	2.340	4.796
Modularity	0.260	0.368	0.66

Based on the betweenness centrality (BC) scores (Supplementary Table S6), OTU3430 (genus: *Ktedonobacter*), OTU3336 (*Mycobacterium*), and OTU1406 (*unclassified_o__Rhizobiales*) were identified as keystone taxa in FL. In group 2, the top-three OTUs were OTU 950 (*norank_f_Anaerolineaceae*), OTU 1204 (*norank_f_MSB-1E8*), and OTU2153 (*norank_f_Gemmatimonadaceae*), while in group 3, the top three OTUs were OTU3155 (*Vicinamibacter*) OTU2808 (*Rhodanobacte*) and OTU2333 (*norank_f_I-10*).

## 4. Discussion

### 4.1. Change of soil properties in different plant communities

Our results found that the soil pH of SSI, SSII, SSIII, and SSIV were weakly alkaline, while that of FL, SSV, and SSVI were weakly acidic (Supplementary Table S3). Studies showed that forests with evergreen broad-leaved plants or both conifer and broad-leaf plants typically have lower pH values (Xie et al., 2015; Lu et al., 2022). The gradual decrease of soil nutrients (SOM, TN, AP, and AN) from sites SSII to SSV suggests that the presence of trees and shrubs consumes more soil nutrients, as reported by Zechmeister-Boltenstern et al. (2015). The evergreen broad-leaf forest community (i.e., SSVI), has more soil nutrients than sites with coniferous forests, possibly due to the higher natural accumulation rate of leaves and branches in the soil.

Soil enzymes are necessary for decomposing organic matter and mineralizing nutrients. They are also key drivers of nutrient supply for plants and may reveal the circulation law of C, P, K, and other nutrients in the soil to reflect the intensity and direction of various biochemical processes (Salazar et al., 2011; Kotroczó et al., 2014). Specifically, catalase mainly helps to decompose hydrogen peroxide, which is related to soil respiration intensity and soil microbial activity (Zheng et al., 2018). Sucrase, urease, and alkaline phosphatase activity usually, respectively, indicate the transformation level of C, N, and P in soil (Li et al., 2019; Cheng et al., 2021a). β-glucosidase participates in the degradation of cellulose in soil and has the potential to monitor biological soil quality (Zang et al., 2018). In our study, there were no apparent differences in the activities of alkaline phosphatase and catalase among the plant communities in all sites (i.e., SSI, SSII, SSIII, SSIV, SSV, and SSVI; Supplementary Table S4). This result reveals the similarity between the rate of P transformation and soil respiration intensity in these plant communities. Site SSII had the highest SOM, TN, AP, and AN content among all the plant communities, possibly due to the *C. coggygria*, which greatly improved the soil nutrient level and quality. The activity of sucrase and β-glucosidase were also the highest in site SSII (Supplementary Table S4), indicating that site SSII had the highest transformation rate of C and soil organic matter. SSII had the second bacterial richness only to that in FL (Table 1). Thus, it is speculated that the root of *C. coggygria* may change the bacteria community or secrete more sucrase and β-glucosidase to promote C and organic matter cycling, consequently increasing soil SOM and TN content. *C. coggygria* could be a pioneer species on the bare soil of KRD regions with a dominant population, as supported by the vegetation survey (Supplementary Table S2; Supplementary Figure S1). However, *C. coggygria* alone cannot dramatically change the environment. In natural ecosystems, plants and microorganisms have a close synergistic effect. The communities of bacteria and plants can not only accelerate the weathering of insoluble minerals in the soil, but also significantly increase the effectiveness of mineral ions in the surface layer of the soil (Liu et al., 2019). Therefore, it is supposed that rhizosphere bacteria could promote the weathering of rocks and provide better conditions for the growth of *C. coggygria*, while the root exudates of *C. coggygria* could change the assembles of bacterial communities (Trivedi et al., 2020). The change in the bacterial community could further alter the composition of the plant community. This mutual reinforcement could be vital in vegetation restoration, and further research will be conducted on the relationship between *C. coggygria* and KRD regions.

### 4.2. Variety of bacterial community composition and structure In different plant communities

The results of UPGMA and Principal Co-ordinates Analysis (PCoA) have revealed a distinct difference in plant communities between FL and the other sites (Figure 3). Moreover, the composition and structure of bacteria in sites SSI, SSII, SSIII, and SSIV were similar, while that of SSV and SSVI were alike. These findings suggest that different plant community compositions can result in distinct evolutionary characteristics for soil bacteria, and that the composition of bacterial communities and plant communities is highly correlated. Specifically, our analysis of soil bacterial communities identified 33 phyla, 77 classes, and 300 families, which were dominated by *Actinobacteria*, *Proteobacteria*, *Acidobacteria*, and *Chloroflexi*. This is consistent with previous studies in the KRD areas (Xue et al., 2017; Qi et al., 2018; Zhu et al., 2019), with the most dominant members of bacterial phyla were the indicator bacteria in sites FL, SSIII, and SSIV (Figure 4; Supplementary Figure S5). However, the most dominant bacterial phyla varied across the study sites, with *Actinobacteria* being the dominant phylum in sites SSI, SSII, SSIII, and SSIV, and *Proteobacteria* in sites SSV and SSVI (Figure 2). The co-occurrence patterns also support our findings (Figure 6). *Proteobacteria* and *Actinobacteria* are copiotrophic bacteria that contribute to increased soil fertility and plant growth (Zheng et al., 2019a; Yang et al., 2021). *Actinobacteria* were the dominant bacteria in sites SSI, SSII, SSIII, and SSIV, where conditions were often more adverse, possibly because they can produce endospores to resist harsh environmental conditions in the KRD areas (Johnson et al., 2007; Yergeau et al., 2010). Studies have also shown that *Actinobacteria* perform well in cave conditions of weathered rock areas in the karst region (Yun et al., 2016; Zhu et al., 2019), and can degrade cellulose and chitin to help release nutrients from plant detritus into the soil. Furthermore, vegetation restoration can increase the relative abundance of *Proteobacteria*, as supported by our results and previous research (Zeng et al., 2017). As the hypothesis mentioned in the introduction, the composition and structure of bacterial communities vary among different plant communities.

Bacterial richness varied significantly among study sites, with the lowest richness observed at site SSI (Table 1). This highlights the crucial role of vegetation in influencing soil bacterial diversity (Xue et al., 2017). Surprisingly, the bacterial richness and diversity at site FL were the highest despite frequent anthropogenic interference. Several studies showed that the microbial communities is highly sensitive to human interference, which can lead to a reduction in microbial community richness and diversity (Bissett et al., 2011; Colombo et al., 2016). However, we found an increase in bacterial diversity in FL. Thus, human impact on microbial communities may vary depending on regions and the degree of interference. In remote mountainous regions, traditional farming practices with moderate disturbance may have created favorable habitats for bacterial communities. However, frequent tillage at hillside fields, especially those with steep slopes fields led soil and water loss, that can ultimately develop into rocky desertification land. This is precisely the reason why the study area has become rocky desertification. The abandoned rocky desertification land, on the other hand, is expected to undergo a process of secondary succession without human interference over time, following the succession sequence of vegetation in the same area. This evolutionary process has been observed in sites SSI, SSII, SSIII, SSIV, SSV, and SSVI (Liang et al., 2015; Song et al., 2019; Zheng et al., 2019b).

### 4.3. Correlations between microbial community and soil properties

The analysis of variation partitioning (VPA) revealed that soil chemical properties have played a significant role in shaping the variance of bacterial community composition (Figure 5). In addition, the results of redundancy analysis (RDA) indicated that TP and TK were the most important nutrient factors influencing the bacterial composition of plant communities in the KRD regions. This finding is consistent with previous studies that suggest soil nutrients are a dominant factor in shaping the structure of the bacterial community (Landa et al., 2014; Deng et al., 2020). Despite the essential role of P and K as necessary elements for plant growth, the bioavailability of phosphorus in karst soils is limited due to the low solubility of phosphate minerals under neutral to alkaline pH values, while the availability of potassium in the karst system of southwest China is restricted by the low carbonate content and high leaching rate under subtropical rainfall conditions (Green et al., 2019). However, when plants grow vigorously, the litter mass they produce can enhance bacterial diversity and abundance in the soil. Moreover, the TP and TK in the soil not only directly impact bacteria but also indirectly influence them by promoting plant growth, which, in turn, affects the bacterial community. Despite these factors, soil P and K are mainly derived from the soil parent material, which, coupled with the poor soil quality, can limit their availability to bacteria. Therefore, these soil nutrient resources are likely to be the primary factors influencing the composition of the bacterial community as a whole.

Our study revealed a significant correlation between soil nutrients and the abundance of dominant bacteria, including *Chloroflexi* and *Actinobacteria*. Notably, we found that the abundance of *Latescibacteria*, *Chloroflexi*, *Gemmatimonadete*, *Actinobacteria*, and *Tectomicrobia* was positively correlated with TK, while the abundance of *Latescibacteria* and *Actinobacteria* was significantly related to TP (Figure 5). These findings highlight the important role of soil P and K in regulating bacterial communities. Moreover, our results suggest that weathering effects in KRD regions play a crucial role in the regulation of soil bacterial communities. Microbial weathering, which involves the direct transformation and migration of mineral elements, is a key process in soil evolution and pedogenesis. Previous studies have also demonstrated the growth-promoting effects of mineral weathering bacteria on plants (Uroz et al., 2011; Cao et al., 2020; Ribeiro et al., 2020; Cheng et al., 2021b). The involvement of *Cyanobacteria* and *Chloroflexi* in rock weathering has been demonstrated (Tang et al., 2012; Tang and Lian, 2012), and this is also in accordance with our sequencing results which have pointed out that the biochemical activities of some microbe have accelerated the mineral elements dissolution and destroyed the mineral structure of the rock surface.

### 4.4. Co-occurrence patterns of bacteria in different plant communities

In natural habitats, species cannot survive independently and they form complex network systems by secreting metabolites and interacting with other microorganisms (Liu et al., 2015; Peura et al., 2015). Our study found that the soil microbial network complexity was highest in sites SSV and SSVI (group 3), followed by SSI, SSII, SSIII, and SSIV (group 2), while the FL (group 1) had the lowest complexity (Figure 6; Table 2). These differences were reflected in factors such as the average clustering coefficient, the average path length, and the modularity index. Our results are consistent with those of Li et al. (2021a) and Morriën et al. (2017), who found that late successional fields had higher soil microbial network complexity than that in early successional fields, while agricultural management could reduce the soil microbial network complexity. Similar to a previous study on the KRD process in southwest China (Jiang et al., 2014), our findings suggest that the lowest network complexity of soil bacteria in farmland (FL) indicates that this type of land requires more management to recover due to its low nutrient content (Supplementary Table S3). Moreover, our observations of serious soil degradation in Chongqing suggest a potential cause of the developing rocky desertification in these karst areas after vegetation destruction. Specifically, excessive use of farmland with only utilization and frequent tillage but little maintenance and management has led to water loss and soil erosion in related regions (Li et al., 2020).

Species with highest betweenness centrality (BC) scores are considered essential for maintaining ecological network connectivity (De Cáceres et al., 2011; Supplementary Table S6). In our study, the top OTU with a high BC score in group 1 (FL) was Ktedonobacter, a mesophilic, aerobic, heterotrophic, and moderately acidophilic bacterium originally isolated from a soil sample of black locust wood in Northern Italy. It has also been found to grow under microaerophilic conditions (Cavaletti et al., 2006). In group 2 (SSI, SSII, SSIII, and SSIV), the top BC score was attributed to *norank_f_Anaerolineaceae*, which is known for its ability to degrade organic matter (Meng et al., 2019). In group 3 (SSV and SSVI), the top BC score was associated with *Vicinamibacter*, which belongs to the K strategy and is able to thrive in undernourished environments. Some members of the Vicinamibacteraceae family have also been found to possess potential chitin-degrading genes (Huber and Overmann, 2018). Our findings suggest that these genera may have played critical roles in maintaining the structure and function of soil bacterial communities in KRD regions. Furthermore, we observed that positive co-occurrence patterns were prevalent in the KRD regions, accounting for more than half of the corresponding links, indicating their primary position in the network.

## 5. Conclusion

We studied soil nutrients, enzyme activities, and bacterial communities in different plant communities in KRD regions. Results showed significant variations in these factors among the plant communities. Herb-and-shrub land (SSII) exhibited the highest contents of SOM, TN, AP, AN and the most active sucrase and β-glucosidase. We speculated that SSII may be the key to positive vegetation succession. The predominant bacterial phylum shifted from *Actinobacteria* (SSI, SSII, SSIII, and SSIV) to *Proteobacteria* (SSV and SSVI). Moreover, total phosphorus (TP) and total potassium (TK) significantly affected soil bacterial community. Co-occurrence network analysis identified key taxa such as *Ktedonobacter*, *norank_f_Anaerolineaceae*, and *Vicinamibacter* that may have played important roles in KRD regions. Finally, the farmland had the highest bacterial richness and diversity, but lowest content of nutrients and enzyme activity due to the water loss and soil erosion caused by poor management. The results could contribute to the development of effective management strategies for KRD areas, promote soil health and ecosystem restoration, and improve the sustainability of restoration efforts.

## Data availability statement

The datasets presented in this study can be found in online repositories. The names of the repository/repositories and accession number(s) can be found at: https://www.ncbi.nlm.nih.gov/, SRP213812.

## Author contributions

LG: conceptualization, methodology, investigation, visualization, and writing—original draft. WW: writing—review and editing. XL: validation, writing—review and editing. XT: formal analysis and software. JY, WZ, and QT: investigation. JW: investigation and writing—review and editing. YL: conceptualization, methodology, funding acquisition, resources, supervision, and writing—review and editing. All authors contributed to the article and approved the submitted version.

## Funding

This work was supported by the Forestry Development Projects through Science and Technology for Chongqing Forestry Bureau (Grant no. 2020-3), the Forestry Development Major Projects through Science and Technology for Chongqing entitled “key techniques and demonstration of integrated application in varieties breeding for colored leaf trees,” and the Graduate Scientific Research and Innovation Foundation of Chongqing (Grant no. CYS19123).

## Conflict of interest

The authors declare that the research was conducted in the absence of any commercial or financial relationships that could be construed as a potential conflict of interest.

## Publisher’s note

All claims expressed in this article are solely those of the authors and do not necessarily represent those of their affiliated organizations, or those of the publisher, the editors and the reviewers. Any product that may be evaluated in this article, or claim that may be made by its manufacturer, is not guaranteed or endorsed by the publisher.

## Supplementary material

The Supplementary material for this article can be found online at: https://www.frontiersin.org/articles/10.3389/fmicb.2023.1180562/full#supplementary-material

## Footnotes

^1^ https://www.arb-silva.de/

^2^ http://huttenhower.sph.harvard.edu/lefse/

^3^ http://www.iplant.cn/
