# Supplementary material for: Soil nutrients, enzyme activities, and bacterial communities in varied plant communities in karst rocky desertification regions in Wushan County, Southwest China
Source: Front Microbiol. 2023 Jun 14;14:1180562. doi: 10.3389/fmicb.2023.1180562 (PMC10301756; doi:10.3389/fmicb.2023.1180562)
Supplement: Supplementary file 1 [file Data_Sheet_1.docx]

Supplementary Material

Soil nutrients, enzyme activities, and bacterial communities in varied plant communities in karst rocky desertification regions in Wushan County, Southwest China

# Supplementary Methods

## Determination of soil enzyme activities

β-glucosidase: 0.025 mL of methylbenzene was added to a soil sample (0.05 g). After 15 min at indoor temperature, the soil sample was incubated with 25 mM *p*-nitrophenyl-β-D-glucopyranoside (0.4 mL) as substrate, in Modified Universal Buffer at pH 6.0 (0.5 mL), at 37 °C. The volume of reaction system was 0.925 mL. After 1 h of incubation, the sample was boiled for 5 min and then filtrated. The released *p*-nitrophenol of filtrate (0.5 mL) was extracted with 0.1 M (Tris-hydroxymethyl-aminomethane)-NaOH (THAM-NaOH) of pH 12.0 (1 mL).

Sucrase: 0.1 g soil sample was added with 0.015 mL of methylbenzene. After 15 min at 37 °C, the soil sample was incubated with 8% sucrose solution (0.75 mL) as substrate, in Phosphate Buffer at pH 5.5 (0.25 mL), at 37 °C for 24 h. The volume of reaction system was 1.015 mL. Following filtration, the filtrate was diluted 10 times with distilled water. Diluted filtrate (0.2 mL) with 3,5-dinitrosalicylic acid (0.5 mL) was boiled for 5 min.

Urease: 0.125 mL of methylbenzene was added to a soil sample (0.25 g). After 15 min at indoor temperature, the soil sample was incubated with 10% urea solution (0.625 mL) as substrate, in Citrate Buffer at pH 6.7 (1.25 mL), at 37 °C for 24 h. The volume of reaction system was 2 mL. Following filtration, the filtrate was diluted 10 times with distilled water. 0.4 mL of diluted filtrate was added with 0.08 mL of sodium phenol solution and 0.06 mL of [sodium](javascript:;) [hypochlorite](javascript:;) [solution](javascript:;). The sample was diluted to 1 mL with distilled water after 20 min at indoor temperature.

Alkaline phosphatase: 0.05 mL of methylbenzene was added to a soil sample (0.1 g). After 15 min shake, the soil sample was incubated with 0.5% phenyl phosphate disodium (0.4 mL, prepared with Borate Buffer at pH 9.4) as substrate at 37 °C for 24 h. Following filtration, 0.05 mL of filtrate was colored with 0.1 mL of 0.3% [aluminum](javascript:;) [sulfate](javascript:;) and 0.02 mL of 2,6-Dibromoquinone-4-chloroimide (prepared with 0.125 g 2,6-Dibromoquinone-4-chloroimide and 10 mL of 96% [ethyl](javascript:;) [alcohol](javascript:;) ) and then diluted to 1 mL with distilled water after the color.

Catalase: the soil sample (0.1 g) was incubated with 30% H_2_O_2_ solution (1 mL) at 25 °C for 20 mins. Then add 0.025 mL of aluminum-potassium alum solution. After centrifugation, take the supernatant and add 0.12 mL concentrated sulfuric acid to prepare the sample for coloring.

The corresponding control was carried out for each sample and enzyme activity by proceeding with the same analytical protocol described, but without the addition of the substrate at the moment of initiating the enzymatic reaction. Each enzyme activity was quantified by reference to a calibration curve incubated with soil under the same conditions described. All soil enzyme activities were determined using a Specord 200 PLUS (Analytik Jena, Jena, Germany) to measure absorbance at different ultraviolet wavelengths (β-glucosidase at 400 nm, sucrose at 540 nm, urease at 630 nm, catalase at 240 nm, and alkaline phosphatase at 660 nm). Specifically, the activity of soil β-glucosidase (β-GC) was expressed by the content of p-nitrophenol (μmol) produced after 24 hours per gram of soil. The activity of soil sucrase (SC) was expressed by the amount of glucose (mg) produced after 24 hours per gram of soil. The activity of soil urease (UE) was expressed by the amount of NH_3_-N (mg) produced after 1h per gram of soil. The activity of soil alkaline phosphatase (AKP) was expressed by the amount of phenol (μmol) produced after 24 hours per gram of soil. The activity of soil catalase (CAT) was expressed in the amount of 1 μmol H_2_O_2_ consumed after 24 hours per gram of soil.

# Supplementary Figures and Tables

## Supplementary Tables

**Supplementary Table S1.** Basic features and evaluations of the various plant communities in the study sites

| Study sites | Type | Altitude  (m) | Location | Typical plant species composition |
| --- | --- | --- | --- | --- |
| FL | Farmed for nearly fifty years | 596  754  695 | 31°4′48″N 109°55′56″E  31°5′1″N 109°56′47″E  31°4′59″N 109°56′12″E | *Ipomoea batatas, Solanum tuberosum* |
| SSⅠ | Herbaceous plants | 455  469  660 | 31°4′59″N 109°55′13″E  31°4′40″N 109°55′25″E  31°4′37″N 109°55′50″E | *Carex brunnea* (D), *Arthraxon hispidus* (A), *Heteropogon contortus* (F) |
| SSⅡ | Shrubs and herbaceous plants | 663  687  675 | 31°4′4″N 109°54′50″E  31°4′10″N 109°55′14″E  31°4′21″N 109°55′42″E | *C. coggygria* (D), *Carex brunnea* (D), *Arthraxon hispidus* (O) |
| SSⅢ | Thickets of shrubs | 365  429  294 | 31°3′23″N 109°54′48″E  31°3′27″N 109°54′23″E  31°3′32″N 109°54′9″E | *C. coggygria* (D), *Myrsine Africana* (A), *Flueggea suffruticosa* (A), *Carex brunnea*(F), *Arthraxon hispidus* (F), etc. |
| SSⅣ | Coniferous forest | 812  576  707 | 31°4′55″N 109°57′11″E  31°5′14″N 109°57′52″E  31°5′5″N 109°57′30″E | *Pinus massoniana* (D), *Cupressus funebris* (F), *C.coggygria* (A), *Wikstroemia micrantha* (O), *Sageretia henryi* (O), *Carex brunnea* (D) |
| SSⅤ | Coniferous broad-leaved mixed forest | 737  663  571 | 31°4′53″N 109°56′52″E  31°4′57″N 109°58′7″E  31°4′58″N 109°58′24″E | *Sapium sebiferum* (D), *Pinus massoniana* (D), *Cupressus funebris* (F), *C. coggygria* (F), *Carex brunnea* (O), etc. |
| SSⅥ | Broad-leaved evergreen forests | 800  629  794 | 31°4′40″N 109°56′17″E  31°4′50″N 109°56′32″E  31°4′56″N 109°57′25″E | *Lithocarpus henryi* (D), *Cupressus funebris* (R), *Myrsine Africana* (D), *C. coggygria* (F), *Carex brunne* (D), *Fluggea suffruticosa* (F), etc. |

NOTE: The abundance of the main plant species is shown in parentheses after each species according to the Clements abundance level. D: Dominant, A: Abundant, F: Frequent, O: Occasional, R: Rare.

**Supplementary Table S2.** Summary of species survey. Farm land (FL), land with only herbs (SSI), herb-and-shrub land (SSII), woody thickets (SSIII), coniferous forest (SSIV), coniferous and broad-leaved mixed forest (SSV), and evergreen broad-leaved forest (SSVI).

| Species | Species type | SSⅠ | SSⅡ | SSⅢ | SSⅣ | SSⅤ | SSⅥ |
| --- | --- | --- | --- | --- | --- | --- | --- |
| *Carex brunnea*  *Arthraxon hispidus*  *Heteropogon contortus*  *Clematis ganpiniana*  *Dendranthema indicum*  *Aster ageratoides*  *Liriope graminifolia*  *Continus coggygria*  *Myrsine Africana*  *Flueggea suffruticosa*  *Abelia chinensis*  *Hypericum monogynum*  *Leptopus chinensis*  *Jasminum floridum*  *Pittosporum truncatum*  *Lespedeza bicolor*  *Wikstroemia micrantha*  *Quercus baronii*  *Viburnum propinquum*  *Cercis chinensis*  *Spiraea wilsonii*  *Cupressus funebris*  *Pinus massoniana*  *Lithocarpus henryi*  *Sapium sebiferum*  *Quercus variabilis*  *Platycarya strobilacea* | Herb  Herb  Herb  Herb  Herb  Herb  Herb  Shrub  Shrub  Shrub  Shrub  Shrub  Shrub  Shrub  Shrub  Shrub  Shrub  Shrub  Shrub  Shrub  Shrub  Tree  Tree  Tree  Tree  Tree  Tree | √  √  √  √  √  √ | √  √  √  √ | √  √  √  √  √  √  √  √  √ | √  √  √  √  √  √  √  √  √  √  √  √ | √  √  √  √  √  √  √  √  √  √  √  √  √  √  √  √  √ | √  √  √  √  √  √  √  √  √  √  √  √  √  √  √  √  √  √  √  √  √  √  √  √ |

**Supplementary Table S3** Soil chemical properties of the varied plant communities

| Study sites | pH | SOM  (g kg^−1^) | Total N  (g kg^−1^) | Total P  (g kg^−1^) | Total K  (g kg^−1^) | Available P  (mg kg^−1^) | Available N  (mg kg^−1^) |
| --- | --- | --- | --- | --- | --- | --- | --- |
| FL | 6.32±0.05c | 6.67±1.61d | 1.08±0.29d | 0.78±0.02c | 5.01±0.15a | 16.03±1.91c | 1.08±0.29d |
| SSⅠ | 7.10±0.05a | 32.41±7.88cd | 3.04±0.69d | 0.84±0.21c | 3.05±0.06b | 6.28±2.41c | 3.04±0.69d |
| SSⅡ | 7.15±0.04a | 136.79±21.22a | 11.29±0.73a | 0.62±0.20c | 2.03±0.14c | 56.98±20.33a | 11.29±0.73a |
| SSⅢ | 7.16±0.02a | 98.06±9.88b | 8.91±0.77b | 0.80±0.07c | 2.03±0.13c | 34.27±6.52b | 8.91±0.77b |
| SSⅣ | 7.26±0.02a | 53.13±3.88c | 5.01±0.14c | 1.87±0.13a | 3.10±0.10b | 16.03±0.53c | 5.01±0.14c |
| SSⅤ | 6.55±0.4b | 21.31±12.50cd | 1.83±0.70d | 1.79±0.49ab | 3.17±0.12b | 5.01±1.65b | 1.83±0.70d |
| SSⅥ | 6.88±0.45ab | 53.02±41.28c | 3.63±4.97d | 1.19±0.81bc | 1.75±0.94c | 7.70±3.21c | 3.63±0.29d |

Note: The same column with different lowercase letters represents significant difference at P<0.05 level using One-way Analysis of variance (One-way ANOVA) analysis.

**Supplementary Table S4** Soil enzyme activities of the varied plant communities

| Study sites | β-GC  μmol/g/24h | SC  mg/g/24h | UE  m/g/24h | AKP  μmol/g/24h | CAT  μmol/g/24h |
| --- | --- | --- | --- | --- | --- |
| FL | 24.44±5.28cd | 3.25±3.14d | 76.94±34.44d | 10.07±1.13b | 14.15±10.27b |
| SSⅠ | 75.18±37.26ab | 41.56±16.17bc | 472.28±286.42c | 35.70±2.90a | 39.19±1.41a |
| SSⅡ | 3.01±1.37d | 94.42±36.31a | 1840.09±86.63a | 29.81±0.65a | 37.74±0.84a |
| SSⅢ | 35.43±18.27bcd | 49.31±12.68bc | 2043.44±35.25a | 32.95±1.26a | 42.34±4.63a |
| SSⅣ | 92.84±45.62a | 53.56±3.04b | 1194.50±30.64b | 36.57±1.24a | 40.57±0.13a |
| SSⅤ | 37.78±26.81bcd | 22.17±18.25cd | 245.33±101.38cd | 26.34±3.18a | 33.58±5.31a |
| SSⅥ | 60.48±31.35abc | 18.53±11.24cd | 361.87±137.44c | 29.75±14.86a | 36.51±0.76ab |

Note: The same column with different lowercase letters represents significant difference at *P*<0.05 level using One-way Analysis of variance (One-way ANOVA) analysis.

**Supplementary Table S5** Welch’s t non-parametric test on bacterial richness and diversity indexes

| Pairwise comparison | OTUs | Shannon | Simpson | ACE | Chao |
| --- | --- | --- | --- | --- | --- |
|  | *P* | *P* | *P* | *P* | *P* |
| FL- SSⅤ | 0.04 | 0.09 | 0.17 | 0.03 | 0.05 |
| FL- SSⅣ | 0.01 | 0.01 | 0.33 | 0.01 | 0.02 |
| FL- SSⅥ | 0.02 | 0.02 | 0.05 | 0.02 | 0.01 |
| FL- SSⅠ | 0.01 | 0.01 | 0.01 | 0.01 | 0.01 |
| FL- SSⅢ | 0.02 | 0.01 | 0.11 | 0.04 | 0.01 |
| FL- SSⅡ | 0.01 | 0.01 | 0.07 | 0.03 | 0.01 |
| SSⅠ- SSⅣ | 0.02 | 0.02 | 0.02 | 0.01 | 0.02 |
| SSⅠ- SSⅢ | 0.03 | 0.07 | 0.05 | 0.01 | 0.02 |
| SSⅠ- SSⅥ | 0.11 | 0.25 | 0.10 | 0.04 | 0.04 |
| SSⅠ- SSⅡ | 0.01 | 0.05 | 0.08 | 0.01 | 0.02 |
| SSⅡ- SSⅣ | 0.02 | 0.20 | 0.02 | 0.11 | 0.43 |
| SSⅡ- SSⅥ | 0.11 | 0.07 | 0.08 | 0.16 | 0.05 |

**Supplementary Table S6** Network indexes of the three groups

| Groups | ID | Genus | Phylum | Betweenness centrality |
| --- | --- | --- | --- | --- |
| Group 1 | OTU3430 | Ktedonobacter | Chloroflexi | 642.76 |
|  | OTU3336 | Mycobacterium | Actinobacteria | 331.05 |
|  | OTU1406 | unclassified_o__Rhizobiales | Proteobacteria | 320.27 |
| Group 2 | OTU950 | norank_f__Anaerolineaceae | Chloroflexi | 1853.18 |
|  | OTU1204 | norank_f__MSB-1E8 | Proteobacteria | 491.13 |
|  | OTU2153 | norank_f__Gemmatimonadaceae | Gemmatimonadetes | 467.81 |
| Group 3 | OTU3155 | Vicinamibacter | Acidobacteria | 3029.11 |
|  | OTU2808 | Rhodanobacter | Proteobacteria | 1765 |
|  | OTU2333 | norank_f__I-10 | Proteobacteria | 1557 |

## Supplementary Figures

**Supplementary Figure S1** (a) Global distribution of karst rocky desertification regions (b) Global distribution of *C. coggygria*, with blue representing China and red representing other countries (c) Distribution of *C. coggygria* and its relationship to karst rocky desertification regions in China.

**Supplementary Figure S2** Rarefaction curves of soil bacteria in different plant communities.

**Supplementary Figure S3** Analysis of similarities (AMOSIM) of bacterial community in different plant communities (based on Bray_Curtis).

**Supplementary Figure S4** (a) Composition of dominant soil bacterial community at class level (Relative abundance > 0.1). (b) One-way ANOVA bar plot on Actinobacteria among the plant communities.

**Supplementary Figure S5** Histogram of LDA scores computed for differentially abundant bacteria in different plant communities identified with a threshold value of 4.0.
