# Supplementary figures and images for: Soil nutrients, enzyme activities, and bacterial communities in varied plant communities in karst rocky desertification regions in Wushan County, Southwest China
Source: Front Microbiol. 2023 Jun 14;14:1180562. doi: 10.3389/fmicb.2023.1180562 (PMC10301756; doi:10.3389/fmicb.2023.1180562)

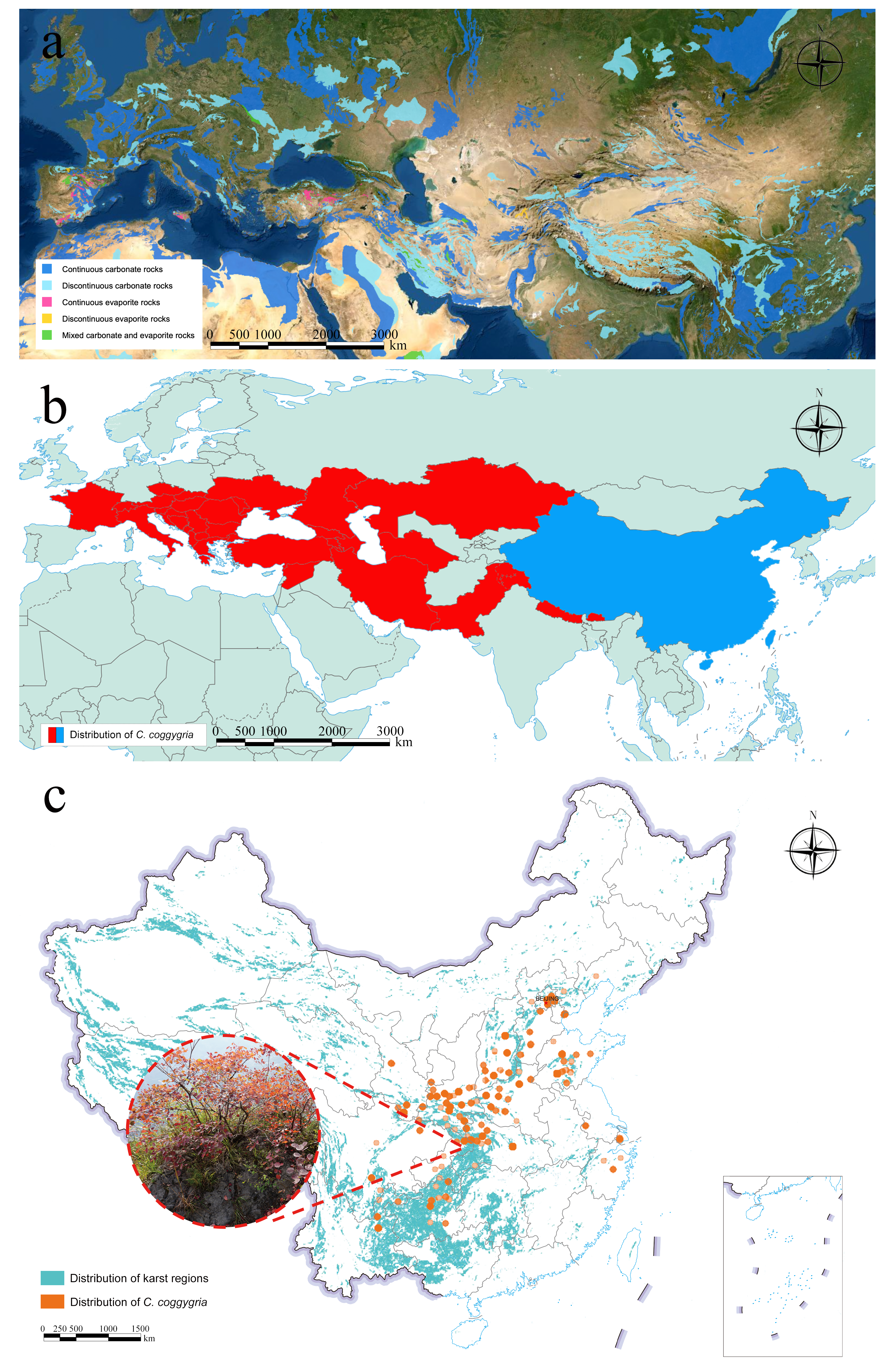

Supplement: Supplementary file 2 [file Image_1.PNG]

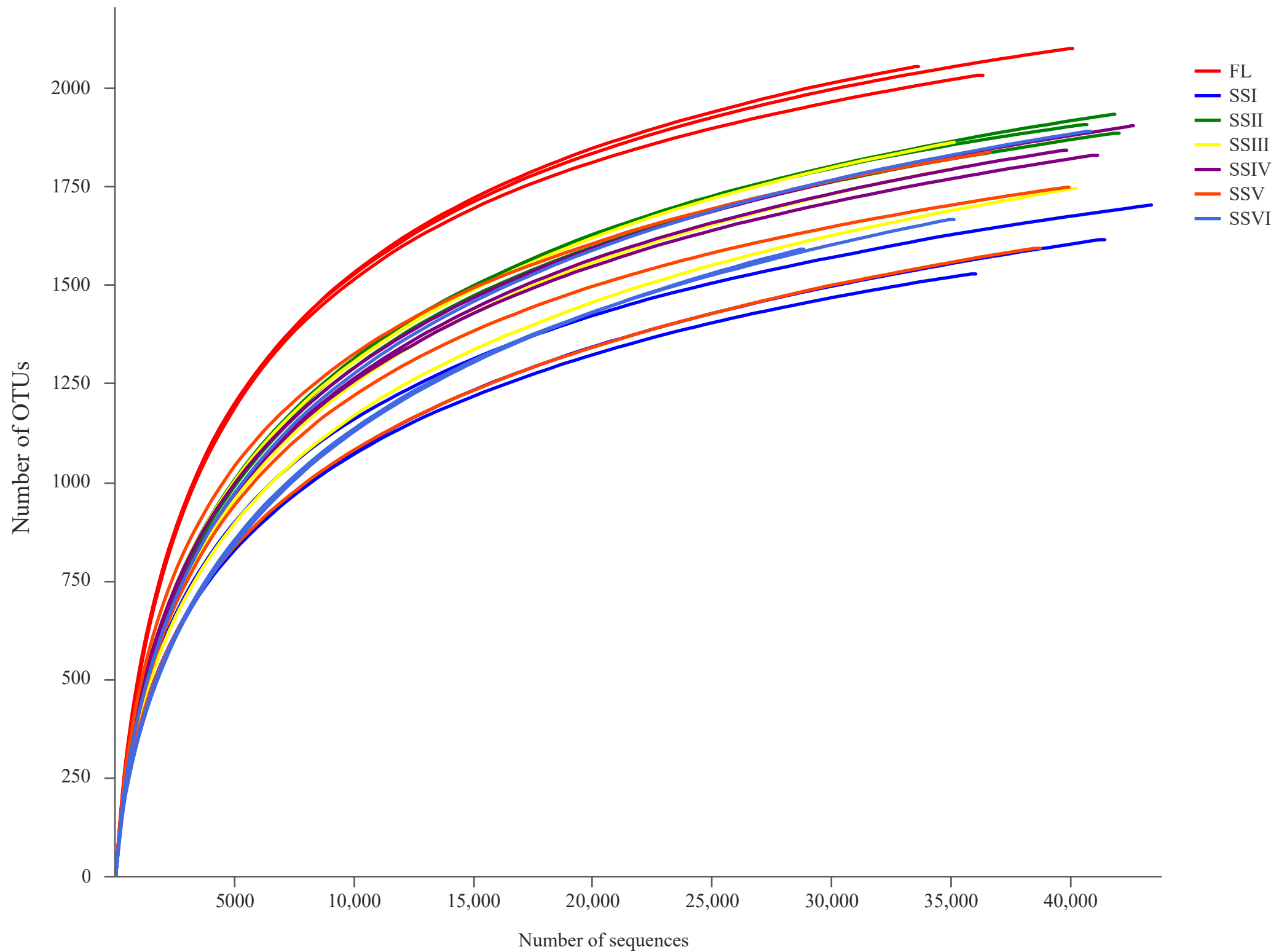

Supplement: Supplementary file 3 [file Image_2.pdf]

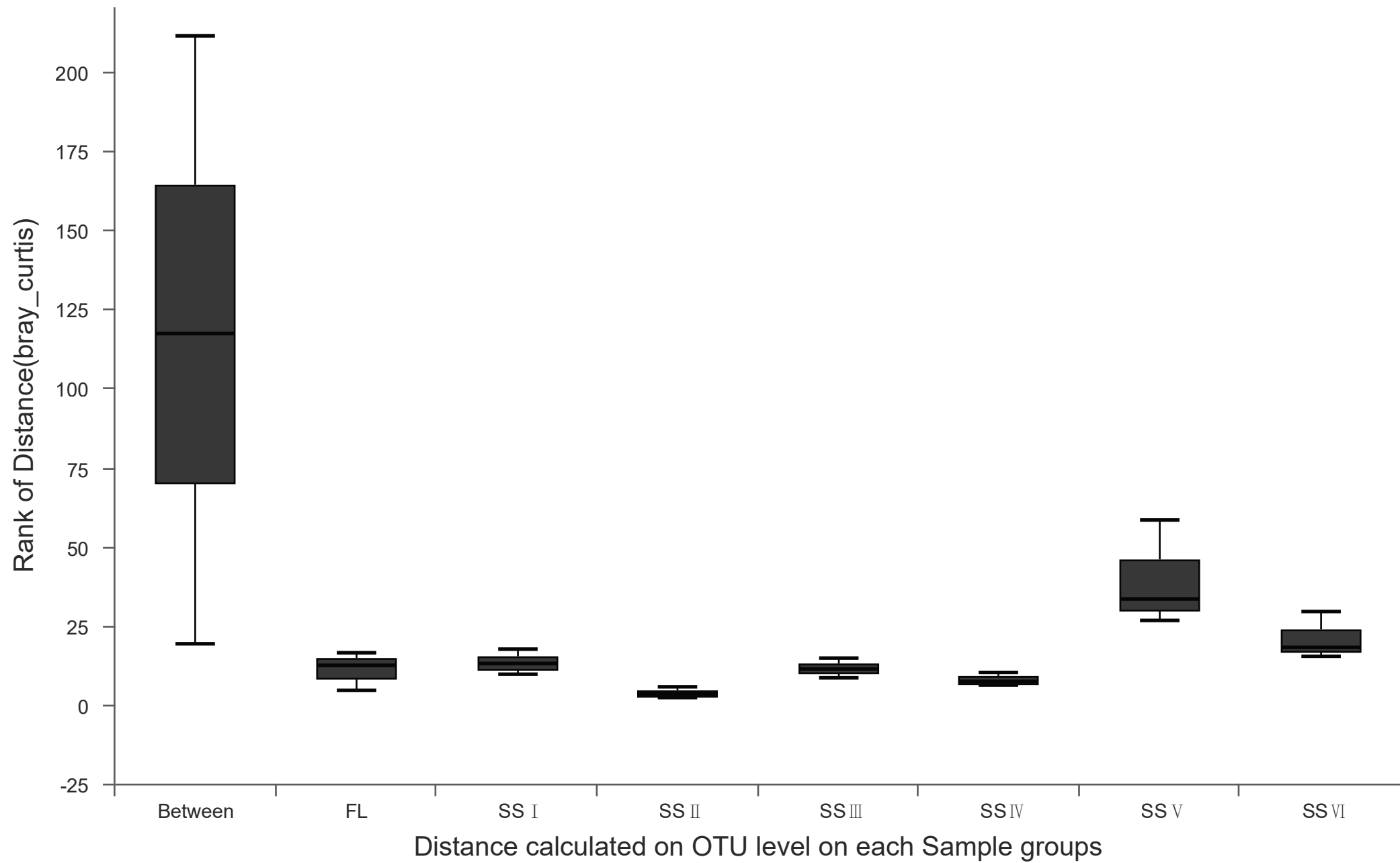

Supplement: Supplementary file 4 [file Image_3.pdf]

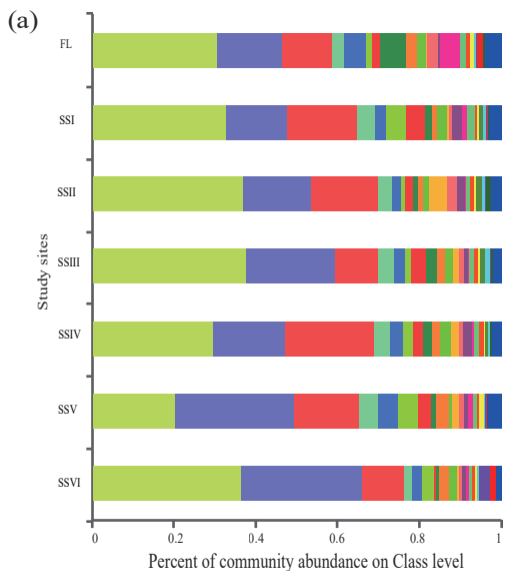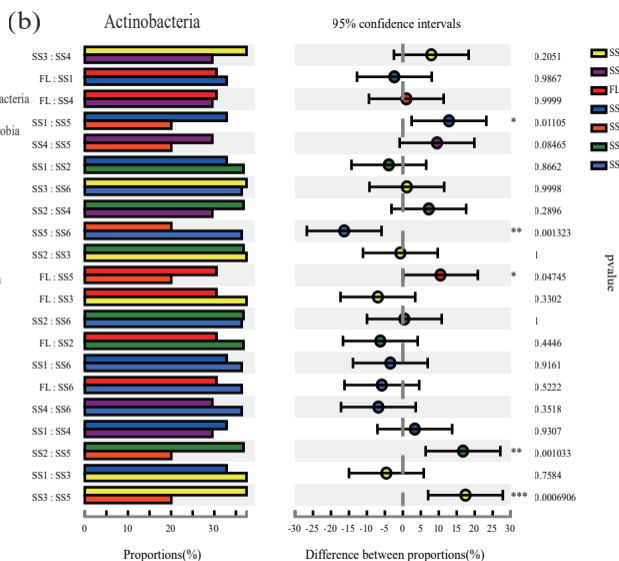

Supplement: Supplementary file 5 [file Image_4.pdf]

LEfSe Bar

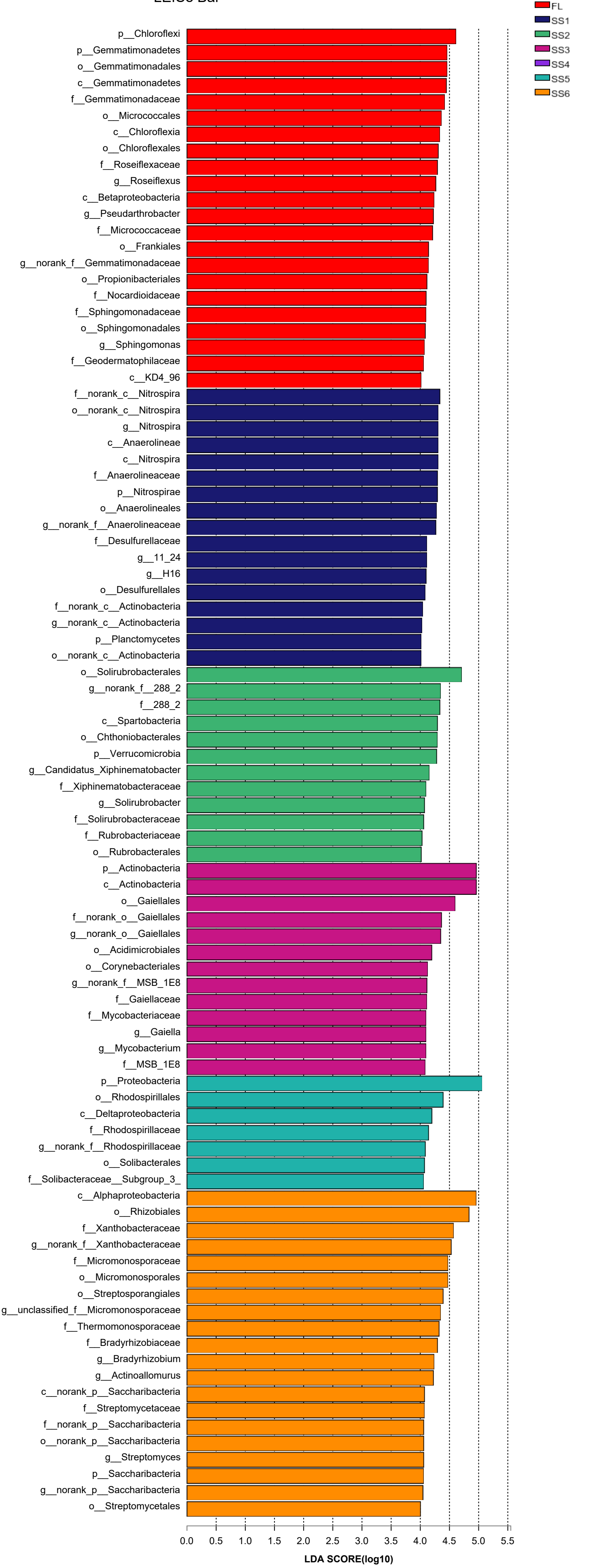

Supplement: Supplementary file 6 [file Image_5.pdf]
